# The Agent-Based Model and Simulation of Sexual Selection and Pair Formation Mechanisms

**DOI:** 10.3390/e20050342

**Published:** 2018-05-04

**Authors:** Rafał Dreżewski

**Affiliations:** AGH University of Science and Technology, Department of Computer Science, 30-059 Cracow, Poland; drezew@agh.edu.pl

**Keywords:** agent-based modeling and simulation, sexual selection, pair formation, speciation, population diversity, entropy, multi-agent systems, evolution

## Abstract

In this paper, the agent-based simulation model of sexual selection and pair formation mechanisms is proposed. Sexual selection is a mechanism that occurs when the numbers of individuals of both sexes are almost identical, while reproduction costs for one of the sexes are much higher. The mechanism of creating pairs allows individuals to form stable, reproducing pairs. Simulation experiments carried out using the proposed agent-based model, and several fitness landscapes were aimed at verifying whether sexual selection and the mechanism of pair formation can trigger sympatric speciation and whether they can promote and maintain population diversity. Experiments were mainly focused on the mechanism of pair formation and its impact on speciation and population diversity. Results of conducted experiments show that sexual selection can start speciation processes and maintain the population diversity. The mechanism of creating pairs, when it occurs along with sexual selection, has a significant impact on the course of speciation and maintenance of population diversity.

## 1. Introduction

Multi-agent systems [1,2,3] and agent-based approach to the construction of software and algorithms [4] are becoming more and more popular among researchers working on modeling and simulation [5,6], as well as researchers dealing with computations [7].

Concepts of *agent* and *multi-agent system* are not strictly defined and they are used by researchers to name various approaches and systems, sometimes very distant from real agent-based systems. In this paper, we will use notions and definitions proposed by Ferber [2]:

- An *agent* is a physical or virtual entity, capable of operating in the environment and able to communicate with other agents.
- In its activities, it strives to achieve its own goals.
- It may possess some resources.
- It may observe the environment, but only locally.
- It possesses only partial knowledge about the environment.
- It has some skills and can offer some services.
- It can also be able to reproduce.

A *multi-agent system* is composed of [2]:

- an environment,
- objects—passive elements of the system,
- agents—active elements of the system,
- relations between the environment, objects and agents,
- operations that allow agents to observe and interact with other system components,
- operators that represent the reactions of other system components to agents’ activities.

On the other hand, biologically and socially inspired artificial intelligence techniques are becoming more and more popular. Such techniques and algorithms include evolutionary algorithms, artificial neural networks, deep neural networks and deep learning, artificial immune systems, ant colony optimization algorithms or swarm algorithms [8,9,10]. These techniques are often used together with the agent-based approach—for example as computational techniques for multi-agent systems [11] or as a set of techniques/algorithms integrated by the agent-based approach, which are used together to solve a given problem (so-called hybrid algorithms) [12].

A connection between the agent-based approach and modeling and simulation can be twofold. First of all, we can create an agent-based model of phenomena of interest, and thus describe them using concepts of agents, environment, and relations. In such a case, we are dealing with agent-based modeling and simulation (ABMS). The second option is to use simulation as a technique supporting the functioning of multi-agent system [6].

The agent-based approach to modeling and simulation provides all fundamental notions and tools that allow creating simulation models of complex real-world phenomena in a very natural way. The agent-based approach allows describing real-world entities and relations using the concepts of autonomous agents, passive objects, environment, and relations between them [5,13,14,15,16].

The ABMS approach is particularly effective in the case of biological mechanisms and phenomena [5,6,13], social phenomena, organizations and relations [6,17,18,19], economic mechanisms and phenomena [15,20], political mechanisms [21], demographic phenomena [22,23], as well as transportation systems and traffic simulations [6].

The ABMS approach can be used when a real-world phenomena result from interactions between autonomous and active entities (agents), passive objects and environment [14]. Usually, these are emergent phenomena, in which some quite complex patterns of behavior of a whole population or society, observed by an external observer, result from simple behaviors of individuals. Emergent phenomena can usually be observed and studied only using computer simulation because it is impossible to predict what would be the results of interactions between many individuals with simple behaviors, even if the results of these simple behaviors, when taken individually, can usually be predicted. Emergent phenomena usually assume the existence of a large population. However, the population does not necessarily have to be very large. The example can be a classical flocking simulation model, in which each individual acts in accordance with the three principles: separation, alignment, and cohesion. In this model, emergent phenomena of flocking exhibits also with small populations (a dozen or several dozens of individuals) and results in a very complex motion and interactions between individuals, which would be extremely hard to recreate using other methods.

The agent-based approach allows modeling of such phenomena in a very natural way and does not require too many simplifications when preparing a simulation model. The ABMS approach enables modeling of entities (agents) with different characteristics and features, taking into account an intelligent behavior of agents and their adaptation to changing environmental conditions. It is also possible to model emergence and disappearance of social relations between agents, emergence and disappearance of organizations and teams (also with learning capabilities at organization/team level), emergence and disappearance of social roles and hierarchies, and shaping and evolution of behavioral norms. Spatial relations are also relatively easy to model because agents and objects are located in an environment with a particular topography and physical dimensions. The agent-based approach also ensures scalability of a simulation model—it is quite easy to extend our model, adding new types of agents, relations, interactions, and resources.

Along with the growing interest in agent-based modeling and simulation, some tools supporting, simplifying and accelerating the process of implementing agent-based simulations appeared. Of considerable importance was the fact that the ABMS approach is often used by researchers from fields far removed from computer science, such as biology, sociology, economics or political science. As a result, systems, programming libraries and tools of varying degrees of sophistication, complexity and putting different requirements regarding the computer science knowledge before the future users were created. The examples of such libraries and systems include NetLogo [16], Repast Suite [15,24], MASON [25,26] and SeSAm [6,27]. A detailed review of agent-based platforms and systems can be found in [28].

The agent-based approach combined with biologically inspired artificial intelligence algorithms can also be used as a computational system. An example of such an approach can be the concept of the evolutionary multi-agent system (EMAS), proposed for the first time in [29]. The EMAS concept is based on a combination of agent-based approach and evolutionary algorithms. In the EMAS approach, a decentralized process of evolution is taking place in an environment of a multi-agent system. A system based on an EMAS approach usually consists of an environment, with agents having an impact on the environment and on each other, resources through which a decentralized process of selection is realized and information, which agents can obtain from each other and the environment. However, agents never have complete knowledge about other elements of the system.

A selection process is carried out thanks to the limited resources, which agents acquire from the environment and each other. These resources are necessary for agents to perform all activities in the system, such as reproduction and migration in the environment in search of better conditions (information, resources or agents ready to reproduce). The resources are obtained from the environment or other agents, in such a way that agents that perform particular tasks better than other agents or are close to achieving a specific goal set for the population acquire more resources. Resources are consumed during the existence of an agent in the system because it executes various types of actions, which, in turn, is associated with the loss of a certain amount of resources. As a result, an agent with low fitness/adaptation rarely reproduces (because a minimum amount of resource is needed for this action), and may even lose the entire resource, which will cause removal of it from the system. In turn, agents with high fitness/adaptation gain many resources, thanks to which they can reproduce more often, and their features (encoded in the genotype) spread with time in the population. The resources are also used to limit the excessive growth of the population—the total amount of resources in the system is constant, and it limits the number of individuals that can exist in the system in a particular moment of time [30].

Recombination and mutation operators are usually identical to those of classical evolutionary algorithms. The main difference from classical evolutionary algorithms is the decentralized selection process (realized with the use of resources) and the fact that agents independently make decisions about executed actions, guided by the level of resources.

The basic EMAS model [29] has been extended to include a possibility of multiple species and sexes existence within the population, and mechanisms of co-evolution and interactions between them [31]. The approach based on a CoEMAS model was applied to many different areas, including multi-modal optimization [32,33], multi-objective optimization [34,35], multi-objective portfolio optimization [36,37,38] and generating investment strategies [39,40]. The sexual selection mechanism for computing systems based on a CoEMAS approach was proposed in [41]. In [42], sexual selection was used as a mechanism of maintaining population diversity in the co-evolutionary multi-agent system for multi-objective optimization.

The model of the multi-agent system with biological and social mechanisms (BSMAS) [43] is a reformulated, and improved version of the model of the co-evolutionary multi-agent system (CoEMAS) proposed in [31]. Compared to a CoEMAS model, the BSMAS model additionally introduces a possibility of defining social relations and structures. It also allows for the consistent integration of agent-based computations, which utilize various biologically and socially inspired artificial intelligence algorithms, as well as agent-based modeling and simulation methods. The BSMAS model will be used in this paper to formally define the simulation model of sexual selection and pair formation mechanism.

In this paper, we focus on agent-based modeling and simulation of sexual selection and pair formation mechanisms and their impact on the species formation processes and increasing and maintaining the population diversity. The speciation models in evolutionary biology can be divided into three groups [44,45]:

- *Allopatric models*—the speciation takes place as a result of the geographic separation of subpopulations, which reduces the flow of genes between subpopulations and ultimately leads to reproductive isolation and emergence of new species.
- *Parapatric models*—subpopulations of primary species live in habitats that only partially overlap, which limits the flow of genes. Such limitation potentially leads to the reproductive isolation and emergence of new species.
- *Sympatric models*—the speciation takes place within the population of primary species only as a result of co-evolutionary interactions with another species or as a result of sexual selection. The selective pressure caused by interactions between co-evolving species or sexes (within a single species) potentially leads to reproductive isolation and emergence of a new species. The space of environment, in which a population is located, does not play any role in the sympatric speciation.

The existence of sexual selection results from two phenomena that occur in natural populations [44,45,46]:

- a cost of reproduction for one of the sexes (usually these are females) is much higher than for the other,
- proportions of both sexes in the population are almost equal.

The above phenomena cause there always being more males than females that are ready for reproduction. As a result, females can choose males based on their particular traits. The males’ traits, which females select, and preferences of females are genetically determined and are inherited by children and further reinforced. As a result, one of the sexes (usually females) evolves towards keeping the reproduction rate at an optimal level, and the sex (usually males) evolves towards increasing the reproduction rate. Such phenomenon leads to “arms races” and co-evolution of sexes.

The authors of [47] proposed a theoretical model of sexual selection with female costs of searching for a partner and variable local carrying capacity of ecosystems. The authors argued that sexual selection maintains, in the long term, the existence of species with overlapping niches and thus maintains the species diversity in an ecosystem.

The agent-based model proposed in this paper also includes a sexual selection mechanism, female costs of searching for a partner and carrying capacity of an ecosystem, which is modeled with the use of limited resources that are needed by agents to perform all of their actions. Additionally, the proposed simulation model includes mechanisms that affect the realism of the simulation, which include male costs of searching for a partner, the mechanism of forming reproducing pairs of agents that live and reproduce together for some time and a spatial structure of an environment. The mechanism of creating reproducing pairs, which significantly reduces the costs of searching for a new partner, was not examined in our previous works [43,48]. The spatial structure of an environment reduces the range of view of agents and thus limits the possibilities of choosing a partner, but it does not have any impact on speciation processes in the proposed model.

The proposed agent-based simulation model shows that sexual selection together with the pair formation mechanism not only maintains population diversity but also leads to speciation—even in small or medium-sized populations, which tend to lose genetic diversity due to genetic drift much quicker than large populations—and thus increases the diversity of ecosystems.

## 2. A Review of Entropy-Based Measures of Population Diversity

In this section, a review of entropy-based measures of population diversity is presented. In addition, entropy-based measures used during experiments are introduced below.

The diversity is one of the fundamental concepts in biology and ecology. As a result of ongoing research, many indicators were proposed to measure the diversity. Unfortunately, the proposed indicators quite often give conflicting and inconsistent results. This fact has prompted some researchers to negate the usefulness of the whole concept of diversity [49].

However, Jost [50] claims not that the very notion of diversity is flawed, but that biologists confuse real diversity with the indicators that are used to measure it. The most commonly used indicators (for example, the Shannon–Wiener index) are entropies, which are rather indicators of the uncertainty of population sampling results than indicators of the diversity of a population itself. In addition, it was shown that most non-parametric diversity indicators are in fact entropies [51,52,53].

Jost [50] claims that direct use of entropy indicators, such as Shannon entropy, does not give us a good measure of the number of species in the population. For example, when there are two ecosystems, the first one with eight equally common species, and the second one with sixteen equally common species, intuition gives us the answer that the second ecosystem should be twice as diverse as the first one. However, the Shannon entropy $x=-\sum_{i=1}^{S}p_{i}\log_{b}\left(p_{i}\right)$, where *S* is the number of species and b=2, gives us the measure of diversity 3.0 for the first ecosystem and 4.0 for the second ecosystem [50].

Of course, it does not mean that Shannon entropy is a weak indicator of diversity—in fact, it is the most insightful and useful of all diversity indicators [50]. It is only relevant not to confuse this indicator with the actual population diversity. To avoid such confusion, Jost [50] proposed a procedure of converting entropy into “true diversity”. He starts from the observation that the diversity indicator creates equivalence classes among various ecosystems. In each of these classes, there is one special ecosystem that has equally common species—its diversity is equal to the number of species. Thus, the problem of finding ecosystem diversity can be reduced to the problem of finding an ecosystem from the same equivalence class that has equally common species. The procedure itself can be summarized as follows [50]: first, the diversity index for *D* equally common species should be calculated, then the resulting expression should be equated to the actual value of the diversity indicator, and the resulting equation should be solved for *D*. The calculated value of *D* would be the “effective number of species”, the “true diversity” of the ecosystem.

When the above procedure is applied to commonly used diversity indicators, the following equations for true diversities will be obtained [50]. The species richness $x\equiv \sum_{i=1}^{S}p_{i}^{0}$, when converted according to the procedure, becomes $D=\sum_{i=1}^{S}p_{i}^{0}$, so, in this case, nothing is changed. Shannon entropy $x\equiv -\sum_{i=1}^{S}p_{i}\ln\left(p_{i}\right)$ is converted to $D=\exp(-\sum_{i=1}^{S}p_{i}\ln\left(p_{i}\right))$. Simpson concentration $x\equiv \sum_{i=1}^{S}p_{i}^{2}$ becomes $D=1/\sum_{i=1}^{S}p_{i}^{2}$.

Most non-parametric diversity indicators—these include species richness, Shannon entropy, Simpson measures, Renyi entropies [54,55], and Tsallis entropies [53]—are monotonic functions of $\sum_{i=1}^{S}p_{i}^{q}$ or limits of such functions, when *q* goes to one [50]. All of these indicators, after conversion using the procedure described above, give the following expression for “true diversity” (also called “Hill numbers”) [50,56]:

(1) $$D\equiv {\left(\sum_{i=1}^{S}p_{i}^{q}\right)}^{1/(1-q)}.$$

Variable *q* is called the “order of diversity”. The order of diversity affects the sensitivity of the indicator to common and rare species. When q=0, the indicator is insensitive to species commonness—in this case, we will obtain the species richness indicator [50]:

(2) $$D_{0}=\sum_{i=1}^{S}p_{i}^{0}.$$

For q=0.5, rare species are preferred and the diversity indicator is defined as follows [50]:

(3) $$D_{0.5}={\left(\sum_{i=1}^{S}\sqrt{p_{i}}\right)}^{2}.$$

For q=1 (neither rare nor common species are preferred), Equation (1) is undefined, but there exists its limit, which is the exponential of Shannon entropy [50]:

(4) $$D_{1}=\exp\left(-\sum_{i=1}^{S}p_{i}\ln\left(p_{i}\right)\right).$$

For q=2, common species are preferred and the following diversity indicator is obtained [50]:

(5) $$D_{2}=1/\left(\sum_{i=1}^{S}p_{i}^{2}\right).$$

The four defined above diversity indicators ($D_{0}$, $D_{0.5}$, $D_{1}$, and $D_{2}$) are used during experiments to measure population diversity (see Section 4.1).

Equation (1) has all properties that we can expect from the correct measure of diversity [50]. It gives the number of species when applied to an ecosystem with equally common species. It will give a doubled value, for any value of the *q* parameter, when a population will be reclassified in such a way that the number of species will be doubled—this is the so-called “doubling” property introduced in [56].

## 3. The Agent-Based Simulation Model of Sexual Selection and Pair Formation Mechanisms

**Figure 1:**
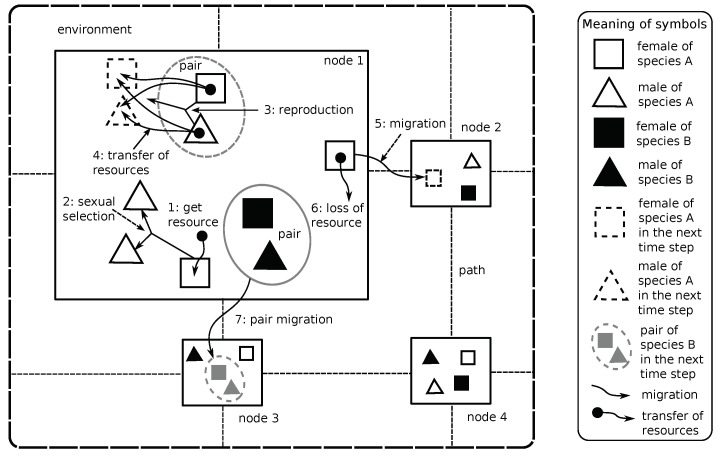
The graphical presentation of the proposed agent-based model. Agents are located within nodes that are connected with paths. In the presented example, there are two species (A and B) and two sexes (females and males) within each species. Every agent tries to get some resources from the environment (1). Females choose partners based on their preferences and male displayed traits (2). Then, a pair is formed, and reproduction takes place (3). During the reproduction, two descendants are created, and resource is transferred from parents to children (4). Agents can migrate between nodes (5). An agent loses some resources during the migration (6). Pairs of agents can also migrate (7).

In the proposed agent-based simulation model, the sympatric speciation takes place as a result of sexual selection and pair formation. Figure 1 illustrates the most important elements of the proposed model, which are explained in the following sections. The formal definition of the model can be found in Appendix A.

### 3.1. Environment

There are two essential elements of every agent-based model—agents and environment (see Figure 1). Agents are always situated within the environment; they can observe and modify it. Other elements of an agent-based model, like resources, information and passive objects, are also situated within the environment. In the proposed model, the environment is composed of nodes connected with paths. The agents are situated within the nodes and can interact only with other agents that are situated within the same node (see Figure 1).

The nodes limit agents’ field of view (see Figure 1). An agent sees only other agents from the same node, and, as a result, it can only interact with such agents. Such mechanism adds the additional level of realism to the proposed model because, in the natural environment, the space factor is always present. There are no populations that exist out of the space of environment, or for which space does not play any role. Even in the case of sympatric speciation, in which space does not play any role because it results only from interactions between species or between sexes, the space factor is always present. The population of a given species is always spread over a certain area, and not concentrated in a single point of space. Individuals always have a limited range of view, so they cannot interact with every single individual from a given population.

In the proposed model, space does not play any role other than limiting the agents’ range of view. Agents can freely migrate between nodes, and there are no obstacles that would prevent their free movement. Thus, the space of environment does not play any role in speciation processes—in Section 4.1, the results of the experiment without the sexual selection and pair formation mechanisms are presented.

### 3.2. Selection

In the proposed model, the role of the environment is not only limited to providing a space in which agents live, but it also evaluates the agents. Every agent tries, from time to time, to gain some resource from the environment. Agents need this resource for performing actions, like reproduction and migration. When an agent is deprived of the resource, it dies, so it is the question of life and death to gain some amount of the resource. The environment uses a fitness function (see Section 3.3) to distribute the resource among agents—the resource is distributed proportionally to the value of agents’ fitness.

The resource is the basis of the selection process in the proposed model. It also limits the carrying capacity of the modeled ecosystem. The total amount of resource in the system is constant. At any time, the resource is in possession of the environment and agents and circulates between them. Agents lose the resource when they perform actions—in such a case, the resource returns to the environment. The environment, in turn, distributes the resource among agents. Such mechanism not only limits the total number of agents that can exist in the system, but also plays a role of a natural selection mechanism.

### 3.3. Fitness Functions

**Figure 2:**
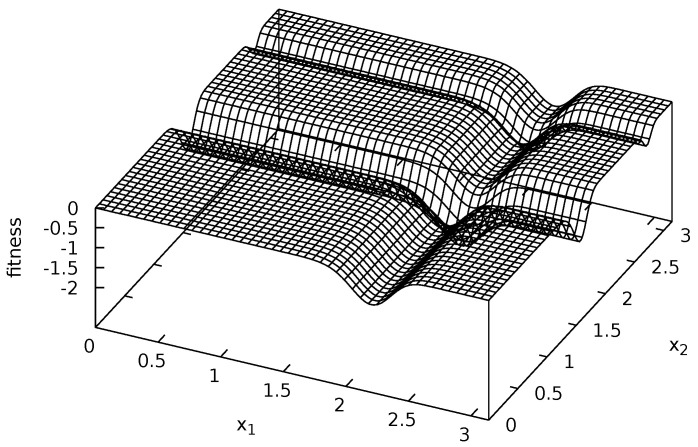
Michalewicz fitness landscape.

**Figure 3:**
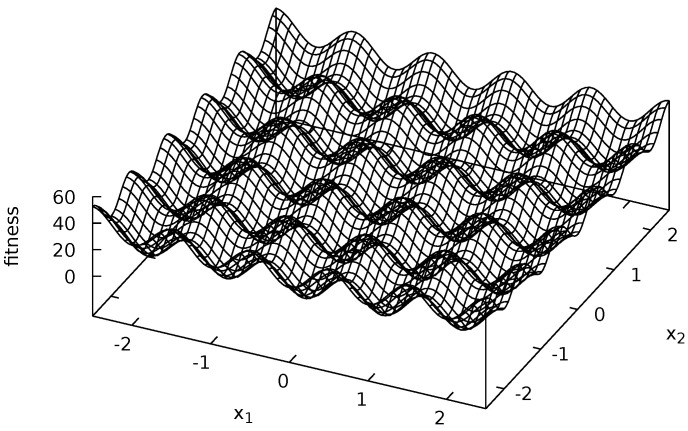
Rastrigin fitness landscape.

**Figure 4:**
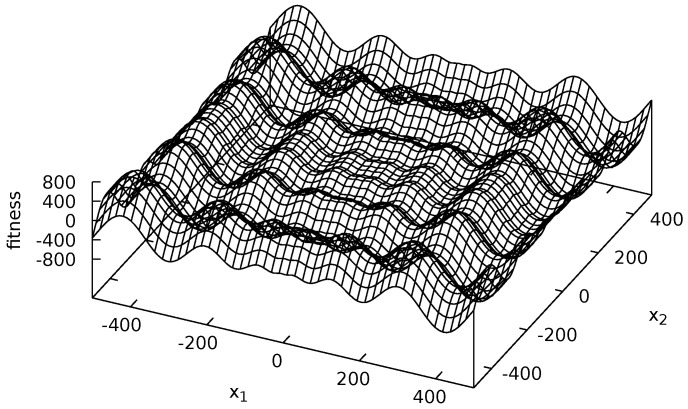
Schwefel fitness landscape.

**Figure 5:**
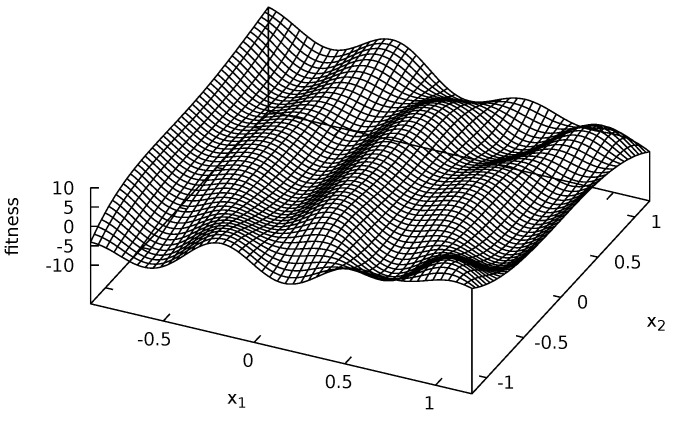
Waves fitness landscape.

Four multi-modal fitness functions were used during experiments: Michalewicz (Figure 2), Rastrigin (Figure 3), Schwefel (Figure 4) and Waves (Figure 5). These functions are models of fitness landscapes—neighborhoods of their local optima can be treated as ecological niches being habitats of various species.

Michalewicz fitness landscape is given by the following equation [57]:

(6) $$\begin{array}{c} f_{1}\left(\vec{x}\right)=-\sum_{i=1}^{n}\left(\sin\left(x_{i}\right)*{\left(\sin(i*x_{i}^{2}/\pi )\right)}^{2*m}\right),\\ x_{i}\in \left[0;\pi \right]\;\mathrm{for}\;i=1,\ldots ,n. \end{array}$$

During experiments, the values of parameters were set as follows: m=10 and n=2. Michalewicz fitness landscape has two local optima, surrounded by deep valleys and flat areas (see Figure 2).

Rastrigin fitness landscape is defined as follows [58]:

(7) $$\begin{array}{c} f_{2}\left(\vec{x}\right)=10*n+\sum_{i=1}^{n}\left(x_{i}^{2}-10*\cos\left(2*\pi *x_{i}\right)\right),\\ x_{i}\in \left[-2.5;2.5\right]\;\mathrm{for}\;i=1,\ldots ,n. \end{array}$$

During experiments, n=2 was assumed. Rastrigin fitness landscape has many regularly spaced local optima (see Figure 3).

Schwefel fitness landscape is defined as follows [58]:

(8) $$\begin{array}{c} f_{3}\left(\vec{x}\right)=\sum_{i=1}^{n}\left(-x_{i}*\sin\left(\sqrt{\left|x_{i}\right|}\right)\right),\\ x_{i}\in \left[-500.0;500.0\right]\;\mathrm{for}\;i=1,\ldots ,n. \end{array}$$

During experiments, n=2 was assumed. Schwefel fitness landscape has many irregularly spaced local optima (see Figure 4).

Waves fitness landscape is given by the following equation [59]:

(9) $$\begin{array}{c} f_{4}\left(\vec{x}\right)=-\left({\left(0.3*x_{1}\right)}^{3}-\left(x_{2}^{2}-4.5*x_{2}^{2}\right)*x_{1}*x_{2}-4.7*\cos\left(3*x_{1}-x_{2}^{2}*\left(2+x_{1}\right)\right)*\sin\left(2.5*\pi *x_{1}\right)\right),\\ x_{1}\in \left[-0.9;1.2\right],\;x_{2}\in \left[-1.2;1.2\right]. \end{array}$$

Waves fitness landscape has many irregularly spaced local optima (see Figure 5).

### 3.4. Agents

Each agent is composed of a genotype, information about the current level of resource possessed, goals (for example “get resource from the environment”, “reproduce” and “migrate to another node”) and actions that are used to realize the goals. There are two different sexes within the population: females and males (see Figure 1)—they differ mainly in a set of actions that they can perform. A female agent can choose a partner for reproduction, and its cost of reproduction is higher than in the case of a male agent (see Figure 1).

The genotype of ag agent $ag^{genotype}=\langle \vec{x},\vec{\sigma }\rangle$ consists of the vector of values of independent variables ($\vec{x}\in D$) and the vector of values of standard deviations used during a mutation ($\vec{\sigma }$), which enable an auto-adaptation of mutation range [60].

In the proposed model, the genotype of ag agent is defined as follows: $ag^{genotype}=\langle \vec{x}=\left[x_{1},x_{2}\right],\vec{\sigma }=\left[\sigma_{1},\sigma_{2}\right]\rangle$. Two independent variables are encoded within the genotype. Two standard deviations are needed to perform a mutation of the genotype (see Section 3.6).

The role of the two independent variables depends on the sex of a given agent. For male agents, the two variables encode displayed traits based on which females make their choice regarding a partner for reproduction. In the case of female agents, the two independent variables encode preferences. A decision of choosing a partner for reproduction is based on a distance in *genetic space* (which has nothing in common with the environment space, in which agents are located) between female preferences (encoded as two independent variables), and male displayed traits (also encoded as two independent variables).

### 3.5. Reproduction, Sexual Selection and Pair Formation

An agent, which is ready for reproduction (a level of its resource is above a certain minimal level), starts looking for a partner. A female agent chooses a partner from male agents that are ready for reproduction and that are located within the same node of the environment as a given female agent (because agents can see each other only within a given node of the environment)—compare Figure 1.

A female agent chooses a partner based on a degree of compliance of preferences (which are encoded in its genotype as two independent variables) with a male agent’s features, which are also encoded in its genotype as two independent variables. The more female’s preferences match male’s features, the higher the probability of selecting a given male agent.

After the selection of a partner is completed, a female agent and a chosen male agent form a pair, and since then they migrate together within the environment and reproduce (see Figure 1). The pair exists for some time, and then it dissolves—this period is configurable in the model. The pair existence helps during the reproduction because agents that form a pair do not have to look for partners (and to lose resources for searching them) when they are ready for reproduction. Both agents are always together in the environment, and reproduction can take place if only both of them are ready for it.

When reproduction occurs, two offspring are created from two parents. During the reproduction, the intermediate recombination [61] and the mutation with self-adaptation [62] are used (see Section 3.6). The generated offspring receive some amount of resource from their parents. The cost of reproduction is higher for a female agent because it gives children more resources than a male agent.

### 3.6. Recombination and Mutation

In the proposed model, the intermediate recombination is used [61]. In the case of this recombination operator, *i*-th value of vector ${\vec{x}}^{a_{l}}$ of $a_{l}$ descendant (agents $a_{j}$ and $a_{k}$ are its parents) is given by [61]:

(10) $$x_{i}^{a_{l}}=\xi_{U(0;1),i}x_{i}^{a_{j}}+\left(1-\xi_{U(0;1),i}\right)x_{i}^{a_{k}}.$$

In Equation (10), $x_{i}^{a_{j}}$ is *i*-th value of vector ${\vec{x}}^{a_{j}}$ of individual $a_{j}$; $x_{i}^{a_{k}}$ is *i*-th value of vector ${\vec{x}}^{a_{k}}$ of individual $a_{k}$; $\xi_{U(0;1),i}$ is the random variable with uniform distribution on the interval (0;1) for *i*-th value of vector ${\vec{x}}^{a_{l}}$.

The intermediate recombination operator is also applied in the case of $\vec{\sigma }$ vector [61]:

(11) $$\sigma_{i}^{a_{l}}=\xi_{U(0;1),i}\sigma_{i}^{a_{j}}+\left(1-\xi_{U(0;1),i}\right)\sigma_{i}^{a_{k}}.$$

During the reproduction, the mutation operator with a mechanism of self-adaptation of its range is also used. Mutation takes place in two stages. First, the parameters of the mutation operator are mutated, and then vector $\vec{x}$ is mutated. The formula specifying a new *i*-th value of vector $\vec{\sigma }$ of ag agent is defined as follows [62]:

(12) $$\sigma_{i}^{\prime }=\sigma_{i}\exp\left(\tau_{0}\xi_{N(0,1)}+\tau \xi_{N(0,1),i}\right).$$

In Equation (12), $\xi_{N(0,1)}$ is the random variable with normal distribution, which has the same value for all elements of vector $\vec{\sigma }$, and $\xi_{N(0,1),i}$ is the random variable with normal distribution for *i*-th value of vector $\vec{\sigma }$.

Recommended values of $\tau_{0}$ and τ parameters are as follows [62]:

(13) $$\begin{array}{c} \tau_{0}=\frac{1}{\sqrt{2n}}, \end{array}$$

(14) $$\begin{array}{c} \tau =\frac{1}{\sqrt{2\sqrt{n}}}. \end{array}$$

In our experiments, n=2 because all of the fitness functions used have two independent variables.

In the second place, the mutation of the vector of independent variables $\vec{x}$ of ag agent is performed, according to the following formula [62]:

(15) $$x_{i}^{\prime }=x_{i}+\sigma_{i}^{\prime }\xi_{N(0,1)}.$$

## 4. The Results of Simulation Experiments

In this section, the results of simulation experiments carried out using the proposed agent-based model of sexual selection with pair formation mechanism are presented. The primary goal of these experiments was to study an impact of the proposed mechanism of pair formation on speciation processes and population diversity. In addition, the ability of sexual selection to initiate speciation processes and maintain population diversity was studied during the experiments.

In all experiments, the developed and implemented multi-agent simulation system based on the proposed spBSMAS model (see Appendix A) was used. The simulation system was implemented in Java and is available under the GNU General Public License.

The values of parameters of spBSMAS model, which were used during the experiments, were as follows:

- maximum age of pair maxPairAge: different values are used during the experiment (0, 1000, 2000, 3000, 4000 and 5000)—the information is provided with the results,
- probability of mutation: mutProb=0.1,
- probability of recombination: recProb=0.8,
- coefficient determining the minimal level of resource that is required for reproduction: minRepRes=0.5,
- coefficient determining how much of the resource is given to offspring by a female agent during reproduction: femaleRepCost=0.4,
- coefficient determining how much of the resource is given to offspring by a male agent during reproduction: maleRepCost=0.2,
- coefficient determining how much of the resource is given back to the environment during migration to another node: migCost=0.05.

The above parameters’ values were obtained during preliminary experiments, which results are not shown in this paper. In the paper, these parameters were set to values that give reasonable results, which are in accordance with real processes taking place in ecosystems. For the sake of clarity of the experimental results, only the values of selected parameters that influence speciation and population diversity are changed during experiments.

### 4.1. Speciation Processes

**Figure 6:**
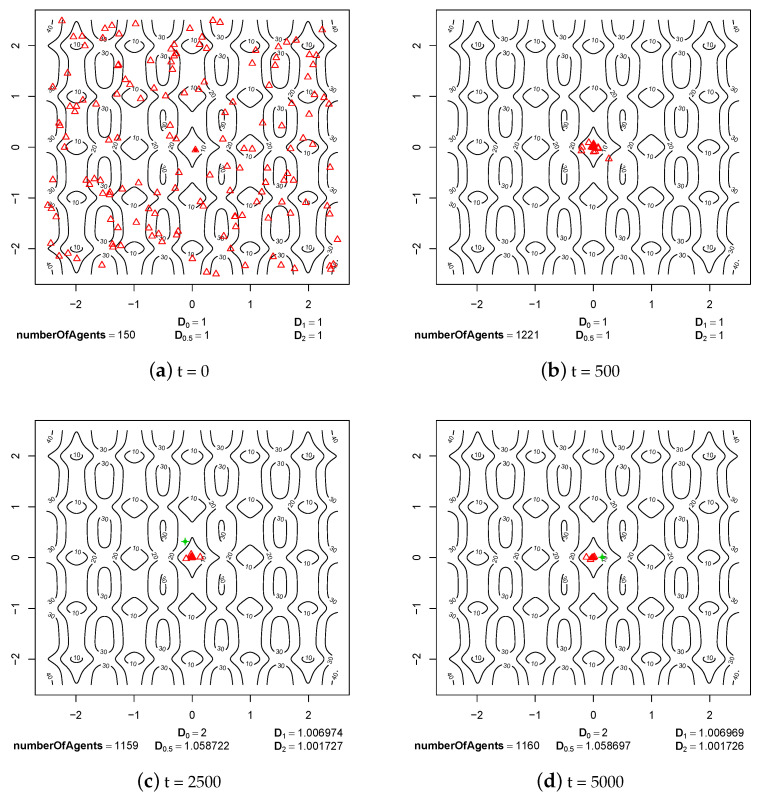
The process of evolution without sexual selection and pair formation mechanisms (*maxPairAge* = 0) in the case of Rastrigin fitness landscape.

**Figure 7:**
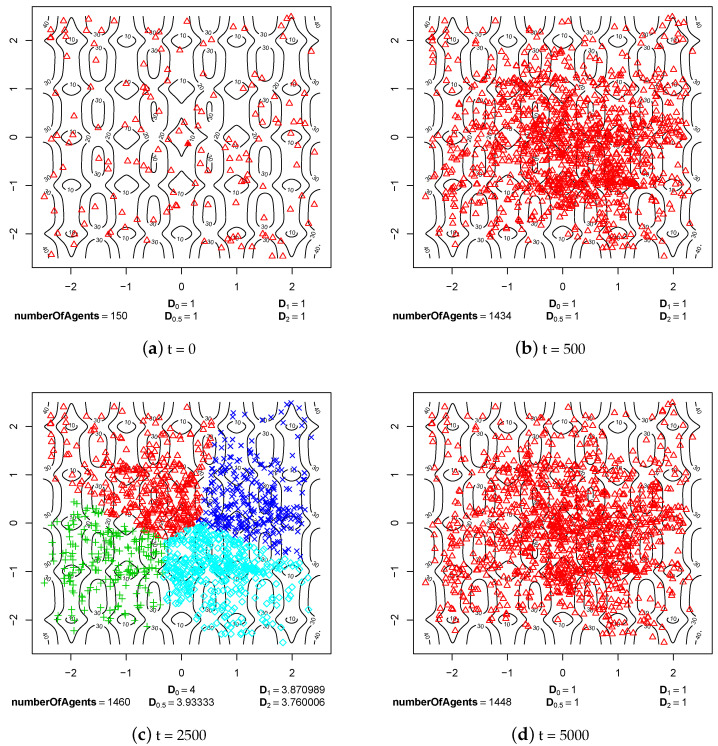
The process of evolution without sexual selection but with the pair formation mechanism turned on (*maxPairAge* = 5000) in the case of Rastrigin fitness landscape.

**Figure 8:**
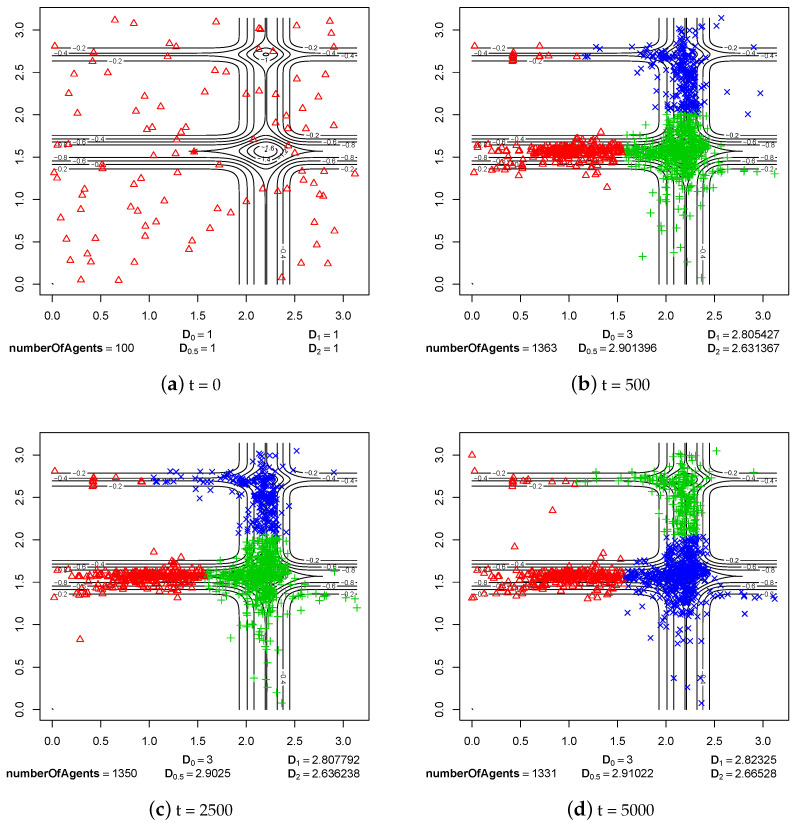
The processes of species formation in the case of Michalewicz fitness landscape and *maxPairAge* = 5000.

**Figure 9:**
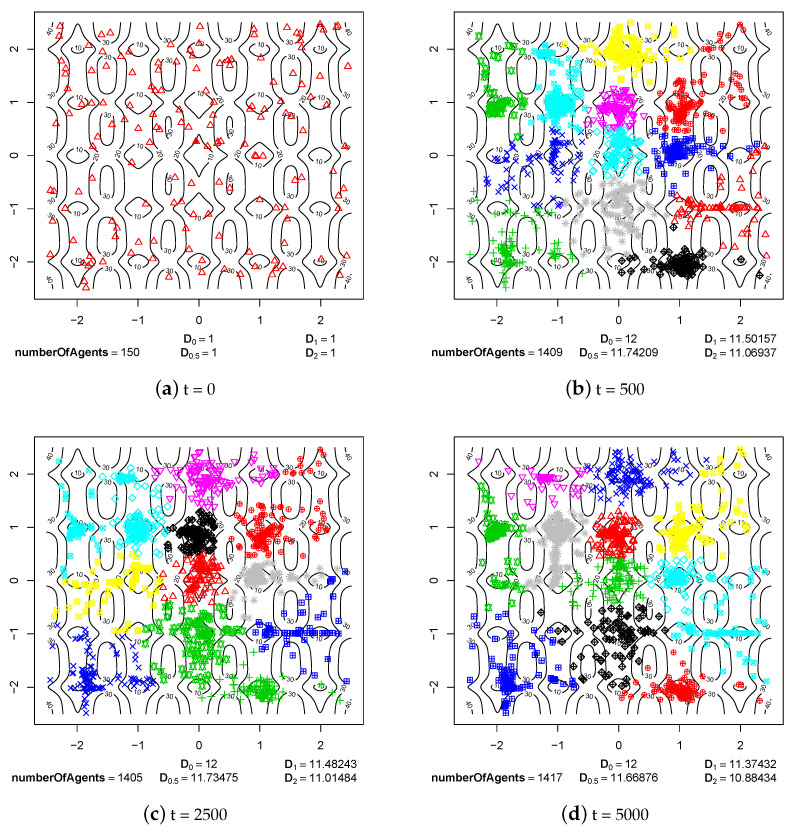
The processes of species formation in the case of Rastrigin fitness landscape and *maxPairAge* = 5000.

**Figure 10:**
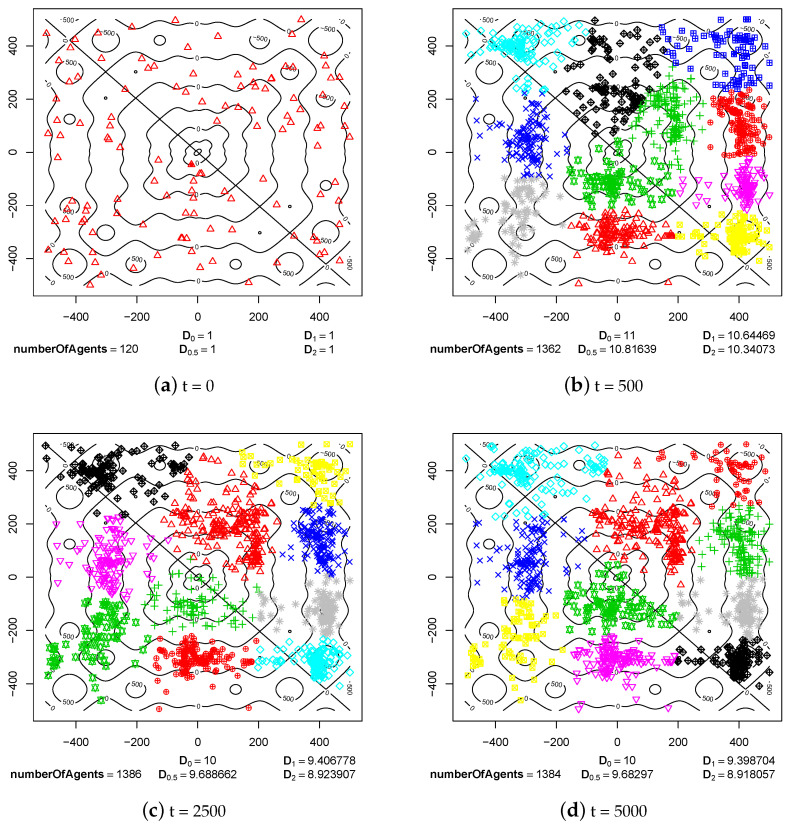
The processes of species formation in the case of Schwefel fitness landscape and *maxPairAge* = 5000.

**Figure 11:**
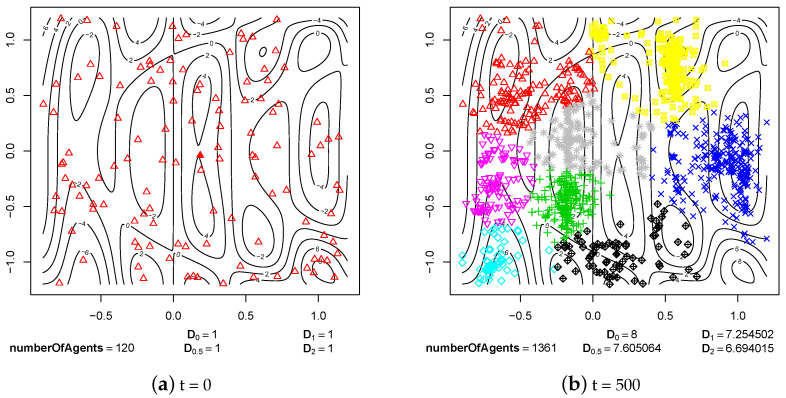

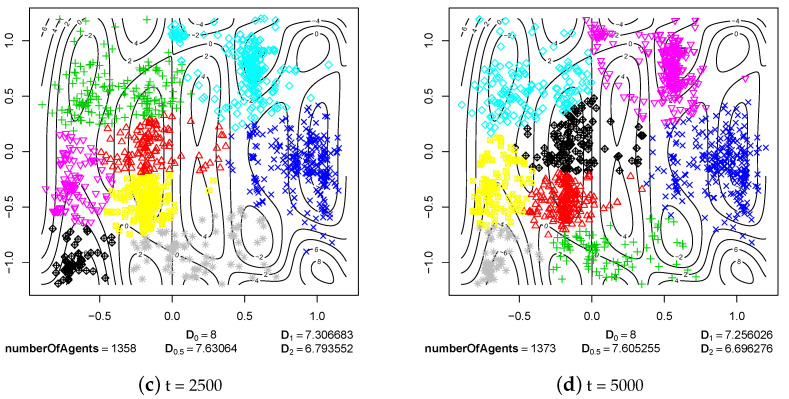
The processes of species formation in the case of Waves fitness landscape and *maxPairAge* = 5000.

The first group of experimental results shows the phenomenon of speciation resulting from sexual selection and pair formation. In Figure 6, Figure 7, Figure 8, Figure 9, Figure 10 and Figure 11, results of typical experiments carried out with the use of different fitness landscapes (Michalewicz, Rastrigin, Schwefel, and Waves) are presented. K-means clustering algorithm based on medoids was used to detect distinct species within the population. The pamk() function from R programming language was used to cluster the population because, in comparison to the pam() function, it provides an optimal number of clusters. Different shapes and colors of points are used to visualize species on fitness contour map—not necessarily the same shapes and colors are used in consecutive steps to identify agents of the same species.

Below each figure, the following information is shown: total number of agents in the population and values of *D* indicator for q=0, q=0.5, q=1 and q=2 (see Equations (2)–(5)).

In Figure 6 and Figure 7, two extreme cases are presented—the case where there is no sexual selection and no pair formation and the case where there is no sexual selection, but individuals can form reproducing pairs.

The process of evolution without sexual selection and pair formation mechanisms is shown in Figure 6. It can be noticed that in this case—especially when compared to the results obtained with sexual selection and pair formation mechanisms turned on, which are presented in Figure 9—the speciation does not take place, and all individuals are located within the same niche. Clustering algorithm detected two subpopulations, but they hardly can be considered as different species because, in fact, they occupy the same niche and are located very close each other in genetic space, so these are rather subpopulations of the very same species. In addition, values of all entropy-based diversity indicators are significantly lower in this case (compare Figure 6 and Figure 9). The lack of sexual selection and pair formation mechanisms lead to complete disappearance of individuals located in areas with lower fitness value. As a result, a highly homogeneous population is obtained at the end of the experiment (see Figure 6d).

In the case of evolution process without sexual selection but with the pair formation mechanism turned on, we can observe that speciation does not take place. However, the individuals are dispersed throughout the whole fitness landscape (see Figure 7). The mechanism of pair formation and the fact that pairs are formed for a whole life of individuals causes that the individuals do not have to search for partners and they can reproduce if only they are ready to do so. As a result, individuals with lower fitness do not disappear from the population. In step 2500, four subpopulations were detected by the clustering algorithm (Figure 7c), but they were not very distinct from each other and quickly disappeared—in step 5000, there is again only one species (Figure 7d).

In all experiments with sexual selection turned on, the value of parameter responsible for time of existence of reproducing pairs was set to maxPairAge=5000 (Figure 8, Figure 9, Figure 10 and Figure 11). The maximal possible value was used because with smaller values speciation processes were not so clearly visible. In such cases, speciation also takes place but sometimes species located in areas with a low fitness value are subjected to extinction.

The phenomenon of disappearance of species results from the fact that, when pairs are dissolved (when they are older than maxPairAge), agents have to search for other partners. This process, however, consumes time and resources. Agents from species located in the areas with a low fitness value collect a much lower amount of resources during their life than agents from species located in the areas with a high fitness value. As a result, a large number of individuals, after dissolving their pairs, will try to find partners by migrating to another vertex, losing lots of resources before possibly finding any partners. Even if such an agent eventually finds a partner, it will have such a small amount of resources that it will not be able to reproduce immediately and it will take some time to collect needed resources again. As a result, a lot of such agents would die before they can reproduce and their species would eventually become extinct.

In the case of Michalewicz fitness landscape (Figure 8), only one species exists at the beginning of experiment (Figure 8a). The phenomenon of speciation can be observed in step 500 of the simulation (Figure 8b). Three distinct species are formed—two of them are located in neighborhoods of local minima, and one is located in a valley. These three species exist stably during consecutive steps of the simulation (Figure 8c,d).

Also in the case of Rastrigin fitness landscape, formed species can be observed in step 500 of the simulation (Figure 9b). The species stably existed until the end of experiment (Figure 9d). As in the case of other experiments, the species were formed as a result of sexual selection—females were choosing partners with features that matched their preferences. In the case of Rastrigin fitness landscape, there were a lot more ecological niches (neighborhoods of local optima), so the whole population was split into many subpopulations that were reproductively isolated.

A very similar situation occurred in the case of Schwefel fitness landscape. Species were formed before step 500 of the simulation (Figure 10b) and their existence was not disturbed until the end of the experiment (Figure 10d). Schwefel fitness landscape is quite similar to Rastrigin landscape and has many ecological niches, so also in this case subpopulations were reproductively isolated from each other. A small flow of genes between subpopulations, which could have appeared because of probabilistic nature of sexual selection mechanism, could not lead to the disappearance of some of the species.

In the case of Waves fitness landscape, processes of speciation are visible in step 500 (Figure 11b). Reproductively isolated subpopulations emerged in most of the neighborhoods of local optima (ecological niches). When we look at consecutive steps of the experiment (Figure 11c,d), it can be easily observed that species exist stably until the end of the experiment. In this case, the possible flow of genes between subpopulations also has not interfered with the existence of species.

### 4.2. Maintaining Population Diversity

**Figure 12:**
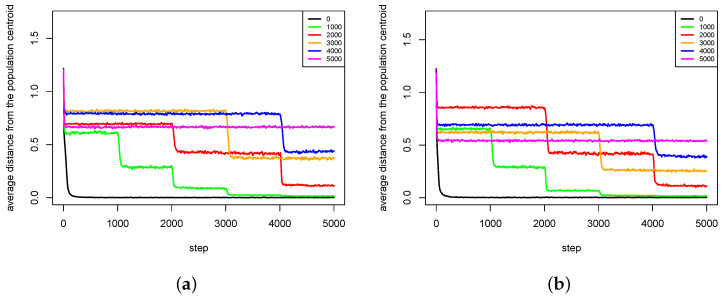
Average distances from the population centroid for different values of maxPairAge parameter. The results of two series (**a**,**b**) of experiments with Michalewicz fitness landscape are presented.

**Figure 13:**
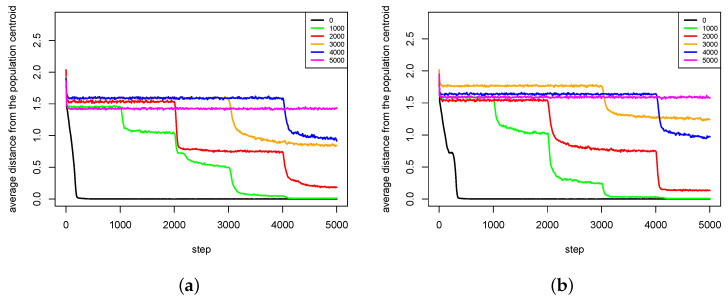
Average distances from the population centroid for different values of maxPairAge parameter. The results of two series (**a**,**b**) of experiments with Rastrigin fitness landscape are presented.

**Figure 14:**
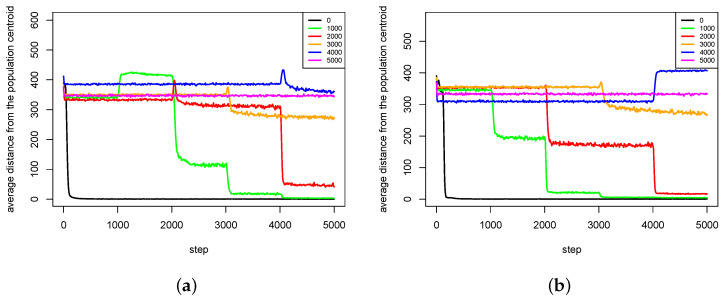
Average distances from the population centroid for different values of maxPairAge parameter. The results of two series (**a**,**b**) of experiments with Schwefel fitness landscape are presented.

**Figure 15:**
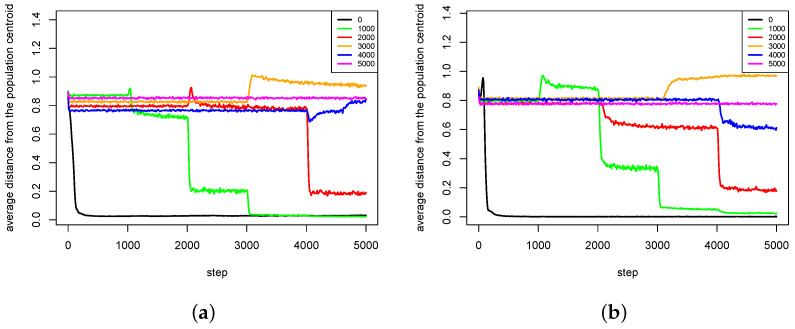
Average distances from the population centroid for different values of maxPairAge parameter. The results of two series (**a**,**b**) of experiments with Waves fitness landscape are presented.

The second group of results (Figure 12, Figure 13, Figure 14 and Figure 15) shows the influence of maximal time of pair existence (maxPairAge) on population diversity. As a measure of population diversity, the average distance of agents from the population centroid was used. The experiments were carried out for the following values of maxPairAge parameter: 0 (no pair formation), 1000, 2000, 3000, 4000 and 5000 (a pair existed until the end of life of one of the agents).

In Figure 12, average distances from population centroid for two experiments with Michalewicz fitness landscape are presented. For every value of maxPairAge parameter, there was a slight decline in population diversity at the very beginning of each experiment. Such phenomenon is caused by the fact that offspring are intensively generated in this period, the population is rapidly growing, and most of the children are genetically close to their parents, so there exists a tendency to form clusters of individuals (subpopulations) in the direct neighborhood of parents. In t=0, the population was dispersed over the whole area, so the population diversity was very high.

The results show that in the case of maxPairAge=5000 population diversity is stably maintained during the whole simulation experiment (Figure 12). Such results are also consistent with the ones presented in Figure 8, which showed that species that were once created remained stable until the end of the experiment. In the case of maxPairAge=0, the population diversity falls dramatically at the very beginning of the experiment and remains at a low level until the end. Such phenomenon is caused by the fact that with maxPairAge=0 pairs are formed, reproduction takes place, but just after that pairs are dissolved, so all agents have to search for new partners for reproduction. As it was stated earlier, such processes weaken the agents—especially those located in the areas of low fitness value. As a result, species found in such areas are subject to extinction.

In the case of maxPairAge=1000, maxPairAge=2000, maxPairAge=3000 and maxPairAge=4000, the scenarios are very similar (Figure 12). When pairs are dissolved, the population diversity falls and stabilizes at a lower level. It does not fall to a very low level because pairs existed stably for some time and their offspring had already the chance to form pairs and can reproduce, so species do not become extinct.

In the case of Rastrigin fitness landscape, (Figure 13), the results are very similar to those obtained with Michalewicz fitness landscape. For maxPairAge=5000, the population diversity is stable throughout the whole experiment. In the case of maxPairAge=0, the population diversity drops at the very beginning of the experiment and remains at a very low level until the end. In the other cases, the population diversity goes down in moments, in which pairs are dissolved.

Much more interesting is the case of Schwefel fitness landscape. It can be clearly observed (Figure 14) that in the case of first series of experiments (Figure 14a) for maxPairAge=1000, maxPairAge=2000, maxPairAge=3000 and maxPairAge=4000, the population diversity firstly increases and then eventually goes down. It turns out that, when pairs were dissolved, agents could quickly find other partners that, however, were genetically distant—female agents were forced to choose male agents, which features did not exactly match their preferences because there were no other male agents. In such a case, their children also differ in genetic terms from parents, so the population diversity rises. In the end, however, the phenomenon of species extinction prevails, and the population diversity decreases.

The second series of experiments is even more interesting (Figure 14b). For maxPairAge=4000, the population diversity significantly increases, and, furthermore, it stays at this level, which is even higher than in the case of maxPairAge=5000. Thus, in this case, the dissolution of pairs caused that speciation process intensified, which resulted in the increased diversity of the population.

In the case of Waves fitness landscape, the results are also very interesting (Figure 15). In the first series of experiments for maxPairAge=1000 and maxPairAge=2000, there is a slight increase of the population diversity in the moment of dissolution of pairs, but after some time the population diversity decreases again (Figure 15a). However, in the case of maxPairAge=3000, the population diversity sharply increases just after the dissolution of pairs and, despite a slight decrease, it stays at a higher level than in the case of maxPairAge=5000.

Even more interesting phenomena can be observed for maxPairAge=4000. At the time of the dissolution of pairs, the population diversity decreases, but it recovers shortly after that and increases to almost the same level as in the case of maxPairAge=5000. It seems that, in this case, agents were able to form pairs again after a very short period, and, furthermore, they were able to form pairs in such configurations that led to the intensification of speciation processes. Because of the lack of other partners, female agents were forced to choose male agents that were not genetically close to them, which caused that offspring were also genetically distant from parents, and speciation processes were intensified. As a result, the population diversity increased. A very similar phenomenon can also be observed in the second series of experiments (Figure 15b) for maxPairAge=3000. Furthermore, in this case, the population diversity increases just after the dissolution of pairs. The population diversity increases to a value higher than in the case of maxPairAge=5000.

From presented results of experiments carried out with the use of the proposed agent-based simulation model, the following conclusions can be drawn:

- Sympatric speciation can be triggered by sexual selection.
- The pair formation mechanism is necessary for a stable existence of species. It reduces the energetic effort necessary for finding a partner for reproduction greatly.
- In the cases when reproduction partners can be found in the nearest neighborhood and the energetic effort needed to find them is not very high, dissolution of existing pairs of agents can intensify speciation processes and increase the population diversity. Such phenomenon occurs because female agents are forced to choose male partners that do not fit their preferences perfectly—simply because there are no other partners in the nearest neighborhood. In such a case, children have much more diverse preferences and features because their parents differ significantly in genetic terms. Such situation can lead to forming new sub-populations, and possibly also new species.

## 5. Conclusions

In this paper, the agent-based simulation model of sexual selection was proposed. In the proposed model, two types of agents exist: female and male. An energetic cost associated with reproduction is higher in the case of female agents, so the agents of this sex choose partners for reproduction from male sex agents.

In the proposed model, agents live within the environment, which distributes resources among agents in proportion to their fitness value. The agents, on the other hand, lose resources while executing actions, such as reproduction and migration. The total amount of resource in the system is constant because it prevents an excessive increase in the number of agents. The model includes the pair formation mechanism, which allows agents from opposite sexes to form pairs. Individuals belonging to a pair can reproduce and move together in the environment.

The primary goal of experiments conducted using the proposed agent-based model was to verify whether sexual selection can trigger sympatric speciation in a population and thus contribute to increasing the population diversity. The second goal was to verify whether the proposed pair formation mechanism can influence somehow the course of speciation processes.

The results of simulation experiments show that, in the proposed agent-based model, sexual selection can start sympatric speciation. Species were formed quite quickly and stably existed during the simulation experiments. The existence of pair formation mechanism had an entirely positive influence on species formation, their stable existence and also on the population diversity. It turned out that the stable existence of species depends strongly on the maximal time of pairs existence (maxPairAge). In most of the experiments, the rule was that a longer maximal time of pairs existence was better for the stable existence of species.

However, the second group of results—in which the population diversity was measured as an average distance of agents from the population centroid—showed a very interesting phenomenon. It turned out that in some experiments with maxPairAge<5000 dissolution of pairs led to the creation of new pairs, which produced very diverse offspring. The diversity of population has even exceeded the level of population diversity obtained for maxPairAge=5000.

Thus, the conclusions from experiments carried out with the proposed agent-based model can be summarized as follows. Sexual selection can trigger sympatric speciation processes in the population. The mechanism of creating pairs is crucial for the stable existence of species because agents do not have to search for new partners and lose their resources steadily. However, when pairs are stable for a long time, there is almost no change in the diversity of the ecosystem. In this case, stagnation can be observed—the diversity is stably maintained. However, new species do not appear. The dissolution of pairs gives a chance that something new and innovative may appear in the population. Pairs are created in new configurations, which eventually results in the emergence of new species and the increase of ecosystem diversity.

The proposed agent-based model can be easily extended with new biological and social mechanisms and interactions. In future research, such mechanisms will be incorporated into the proposed model to make it more realistic and allow the emergence of new phenomena. In addition, based on BSMAS approach, new agent-based models of ecosystems will be proposed, which will include more complex biological and social relationships between species, sexes, and groups of agents.

## Appendix A. The Agent-Based Model of Evolving Population with Sexual Selection and Pair Formation Mechanisms

### Appendix A.1. Agent-Based Simulation System

The agent-based simulation system with sexual selection and the pair formation mechanisms is defined as follows:

(A1) $$\begin{array}{cc} \mathrm{spBSMAS}(t)= & \langle EnvT\left(t\right)=\left\{et\right\},Env\left(t\right)=\left\{env\right\},ElT\left(t\right)=VertT\left(t\right)\cup ObjT\left(t\right)\cup AgT,\\ & \;ResT\left(t\right)=\left\{rt\right\},InfT\left(t\right)=\emptyset ,Rel\left(t\right),Attr\left(t\right)=\left\{age,genotype\right\},Act\left(t\right)\rangle . \end{array}$$

The above symbols have the following meaning:

- EnvT(t) is the set of environment types in time *t*;
- Env(t) is the set of environments in time *t*;
- ElT(t) is the set of types of elements that can exist within the system in time *t*;
- $VertT\left(t\right)=\left\{vt\right\}$ is the set of types of vertices that can exist within the system in time *t*;
- ObjT(t)=∅ is the set of object (passive elements) types that can exist within the system in time *t*;
- $AgT\left(t\right)=\left\{female,male\right\}$ is the set of agent types that can exist within the system in time *t*;
- ResT(t) is the set of resource types that can exist within the system in time *t*, the amount of resource of type rest(t)∈ResT(t) will be denoted as $r^{rest}\left(t\right)$;
- InfT(t) is the set of information types that exist in the system, the information of type inft(t)∈InfT(t) will be denoted as $inf^{inft}\left(t\right)$;
- Rel(t) is the set of relations between sets of agents, objects, and vertices;
- Attr(t) is the set of attributes of agents, objects, and vertices—the are two possible attributes: age and genotype of an agent;
- Act(t) is the set of actions that can be executed by agents, objects, and vertices.

The set of actions is defined as follows:

(A2) $$\begin{array}{cc} Act= & \{die,get\_resource,ready\_for\_reproduction,choose,reproduce,\\ & \;create\_pair,dissolve\_pair,migrate,give\_resource\}. \end{array}$$

### Appendix A.2. Environment

Environment type et is defined in the following way:

(A3) $$et=\langle EnvT^{et}=\emptyset ,VertT^{et}=VertT,ResT^{et}=ResT,InfT^{et}=\emptyset \rangle .$$

The above symbols have the following meaning:

- $EnvT^{et}\subseteq EnvT$ is the set of environment types that may be connected with environment of type et.
- $VertT^{et}\subseteq VerT$ is the set of types of vertices that may exist within the environment of type et.
- $ResT^{et}\subseteq ResT$ is the set of resource types that may exist within the environment of type et.
- $InfT^{et}\subseteq InfT$ is the set of information types that may exist within the environment of type et.

Environment env of type et is defined as follows:

(A4) $$env=\langle gr^{env},Env^{env}=\emptyset \rangle .$$

Directed graph $gr^{env}$ is defined as follows:

(A5) $$gr^{env}=\langle Vert,Arch,cost\rangle .$$

The above symbols have the following meaning:

- Vert is the set of vertices.
- Arch is the set of arches.
- Function cost computes an amount of resource, that an agent migrating between two nodes would lose.

A distance between two nodes is defined as the length of the shortest path between them in graph $gr^{env}$. $Env^{env}\subseteq Env$ is the set of environments of types belonging to EnvT that are connected to environment env.

Vertex type vt is defined in the following way:

(A6) $$\begin{array}{cc} vt= & \langle Attr^{vt}=\emptyset ,Act^{vt}=\left\{give\_resource\right\},ResT^{vt}=ResT,\\ & \;InfT^{vt}=\emptyset ,VertT^{vt}=VertT,ObjT^{vt}=\emptyset ,AgT^{vt}=AgT\rangle . \end{array}$$

The above symbols have the following meaning:

- $Attr^{vt}\subseteq Attr$ is the set of attributes of vt vertex at the beginning of its existence;
- $Act^{vt}\subseteq Act$ is the set of actions, which vertex vt can execute at the beginning of its existence, when asked for it;
- $ResT^{vt}\subseteq ResT$ is the set of resource types, which can exist within vertex vt at the beginning of its existence;
- $InfT^{vt}\subseteq InfT$ is the set of information, which can exist within vertex vt at the beginning of its existence;
- $Vt^{vt}$ is the set of types of vertices that can be connected with vertex vt at the beginning of its existence;
- $ObjT^{vt}\subseteq ObjT$ is the set of types of objects that can be located within vertex vt at the beginning of its existence;
- $AgT^{vt}\subseteq AgT$ is the set of types of agents that can be located within vertex vt at the beginning of its existence;
- give_resource is the action of giving a certain amount of resource to an agent.

Vertex vert∈Vert of type $vt\in VertT^{env}$, which is a basic element of the environment, is defined as follows:

(A7) $$\begin{array}{cc} vert= & \langle Attr^{vert}=\emptyset ,Act^{vert}=Act^{vt},Res^{vert}=\left\{r^{vert}\right\},\\ & \;Inf^{vert}=\emptyset ,Vert^{vert},Obj^{vert}=\emptyset ,Ag^{vert}\rangle . \end{array}$$

The above symbols have the following meaning:

- $Attr^{vert}\subseteq Attr$ is the set of attributes of vertex vert—this set can change during a vertex’s lifetime;
- $Act^{vert}\subseteq Act$ is the set of actions, which vertex vert can execute, when asked for it—this set can change during a vertex’s lifetime;
- $Res^{vert}$ is the set of resources of types from ResT set;
- $Inf^{vert}$ is the set of information of types from InfT set;
- $Vert^{vert}$ is the set of types of vertices from VertT set that are connected with vertex vert;
- $Obj^{vert}$ is the set of objects of types from ObjT set that are located in vertex vert;
- $Ag^{vert}$ is the set of agents of types from AgT set that are located in vertex vert.

$r^{vert}$ is the amount of resource of type rt that is possessed by vertex vert. $Vert^{vert}$ is the set of four vertices connected with vertex vert (see Figure 1). $Ag^{vert}$ is the set of agents located in vertex vert.

### Appendix A.3. Agents

There are two types of agents in the system: female and male. These two types of agents and their activities during the lifetime are defined in the sections below.

#### Appendix A.3.1. Female Agent

The female type of agent is defined in the following way:

(A8) $$\begin{array}{cc} female= & \langle Gl^{female}=\left\{gl_{1},gl_{2},gl_{3}\right\},Attr^{female}=\left\{age,genotype\right\},Act^{female}=\{die,get\_resource,\\ & \;choose,reproduce,create\_pair,dissolve\_pair,migrate\},ResT^{female}=ResT,\\ & \;InfT^{female}=\emptyset ,ObjT^{female}=\emptyset ,AgT^{female}=\emptyset \rangle . \end{array}$$

The above symbols have the following meaning:

- $Gl^{female}$ is the set of goals of female agent at the beginning of its existence;
- $Attr^{female}\subseteq Attr$ is the set of attributes of a female agent at the beginning of its existence, age is the current age, genotype contains the encoded female preferences (two independent variables) and the parameters of mutation (standard deviations)—compare Section 3.4 and Section 3.6;
- $Act^{female}\subseteq Act$ is the set of actions, which a female agent can execute at the beginning of its existence;
- $ResT^{female}\subseteq ResT$ is the set of types of resources, which can be used by a female agent at the beginning of its existence;
- $InfT^{female}\subseteq InfT$ is the set of information types, which can be used by a female agent at the beginning of its existence;
- $ObjT^{female}\subseteq ObjT$ is the set of types of objects that can be located within a female agent at the beginning of its existence;
- $AgT^{female}\subseteq AgT$ is the set of types of agents that can be located within a female agent at the beginning of its existence.

The set of goals $Gl^{female}$ includes the following goals:

- $gl_{1}$ is the goal of getting a certain amount of resource from the environment;
- $gl_{2}$ is the goal of reproducing;
- $gl_{3}$ is the goal of migrating to another vertex.

The actions have the following meaning:

- die is the action of death—an agent dies when it runs out of resources.
- get_resource is the action of getting a certain amount of resource from the environment.
- choose is the action of choosing a partner for reproduction from a set of male agents that are located within the same vertex and are ready for reproduction (that is, which executed ready_for_reproduction action).
- reproduce is the action of reproducing with the use of recombination and mutation operators. A female agent executes this action when it is ready for reproduction, and a male partner was chosen (with the use of choose action), or a female agent is already paired with a male agent. During the reproduction, a female agent gives a certain amount of resources to its offspring.
- create_pair is the action of forming a pair with a selected male agent.
- dissolve_pair is the action of dissolving a pair if it is older than maxPairAge.
- migrate is the action of migrating to another vertex in search of resources or partners for reproduction.

The pseudocode of activities of the female agent during its lifetime is presented in Algorithm 1. Parameter femaleRepCost determines how much of the resource is given to the offspring. The migCost parameter determines how much of the resource is given back to the environment during migration to another node.

**Algorithm 1:** The pseudocode of activities of the female agent.

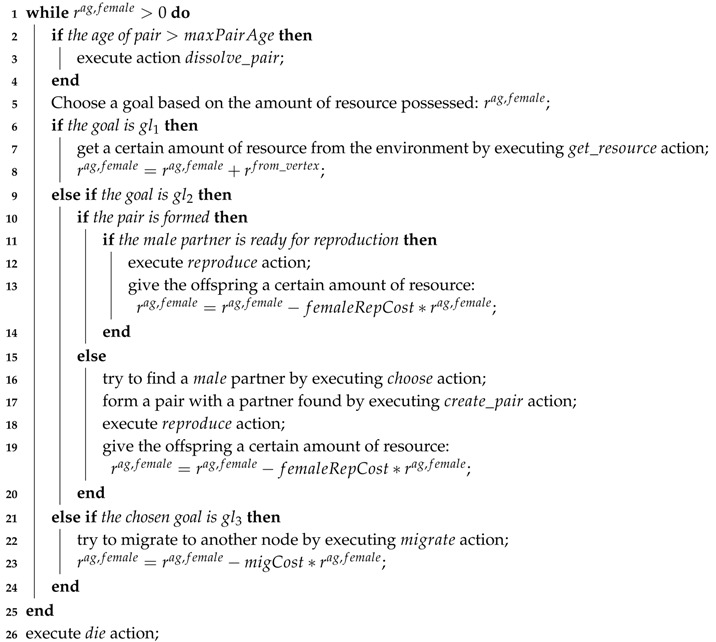

The female agent is defined in the following way:

(A9) $$\begin{array}{cc} ag^{female}= & \langle Gl^{ag,female}=Gl^{female},Attr^{ag,female}=Attr^{female},Act^{ag,female}=Act^{female},\\ & Res^{ag,female}=\left\{r^{ag,female}\right\},Inf^{ag,female}=\emptyset ,Obj^{ag,female}=\emptyset ,Ag^{ag,female}=\emptyset \rangle . \end{array}$$

The above symbols have the following meaning:

- $Gl^{female}$ is the set of goals, which the female agent tries to realize—this set can change during the agent’s lifetime;
- $Attr^{female}\subseteq Attr$ is the set of attributes of the female agent—this set can change during the agent’s lifetime;
- $Act^{female}\subseteq Act$ is the set of actions, which the female agent can execute in order to realize its goals—this set can change during the agent’s lifetime;
- $Res^{female}$ is the set of resources (of types from ResT set) which are used by the female agent;
- $Inf^{female}$ is the set of information (of types from the InfT set), which the female agent can possess and use;
- $Obj^{female}$ is the set of objects (of types from the ObjT set), that are located within the female agent;
- $Ag^{female}$ is the set of agents (of types from the AgT set), that are located within the female agent.

The notation $Gl^{ag,female}$ means “the set of goals of the female type agent ag”. $r^{ag,female}$ is the amount of rt type resource that is possessed by agent $ag^{female}$.

#### Appendix A.3.2. Male Agent

The male type of agent is defined in the following way:

(A10) $$\begin{array}{cc} male= & \langle Gl^{male}=\left\{gl_{1},gl_{2},gl_{3}\right\},Attr^{male}=\left\{age,genotype\right\},Act^{male}=\{die,get\_resource,\\ & ready\_for\_reproduction,reproduce,create\_pair,dissolve\_pair,migrate\},\\ & ResT^{male}=ResT,InfT^{male}=\emptyset ,ObjT^{male}=\emptyset ,AgT^{male}=\emptyset \rangle . \end{array}$$

The meaning of all the above symbols is the same as in the case of female type agent—compare Equation (A8). genotype contains the encoded male features (two independent variables) and the parameters of mutation (standard deviations)—compare Section 3.4 and Section 3.6.

The set of goals $Gl^{male}$ is identical as in the case of female type agent and includes the following goals:

- $gl_{1}$—getting resource from the environment,
- $gl_{2}$—reproducing,
- $gl_{3}$—migrating to another vertex.

The actions have the following meaning:

- die is the action of death—an agent dies when it runs out of resources.
- get_resource is the action of getting a certain amount of resource from the environment.
- ready_for_reproduction is the action executed when a given male agent is ready for reproduction. Then, a female agent that is also ready for reproduction chooses the male agent (by executing choose action) or a female agent that is paired with the male agent, and is also ready for reproduction, executes reproduce action.
- reproduce is the action of reproducing (with the use of recombination and mutation operators)— a male agent executes this action when it is ready for reproduction and has a female partner.
- create_pair is the action of forming a pair with a female agent.
- dissolve_pair is the action of dissolving a pair if it is older than maxPairAge.
- migrate is the action of migrating to another vertex.

The pseudocode of activities of the male agent during its lifetime is presented in Algorithm 2. Parameter maleRepCost determines how much of the resource is given to the offspring. The migCost parameter determines how much of the resource is given back to the environment during migration to another node.

The male agent is defined in the following way:

(A11) $$\begin{array}{cc} ag^{male}= & \langle Gl^{ag,male}=Gl^{male},Attr^{ag,male}=Attr^{male},Act^{ag,male}=Act^{male},\\ & \;Res^{ag,male}=\left\{r^{ag,male}\right\},Inf^{ag,male}=\emptyset ,Obj^{ag,male}=\emptyset ,Ag^{ag,male}=\emptyset \rangle . \end{array}$$

The notation $Gl^{ag,male}$ means “the set of goals of the male type agent ag”. $r^{ag,male}$ is the amount of rt type resource that is possessed by agent $ag^{male}$.

**Algorithm 2:** The pseudocode of activities of the male agent.

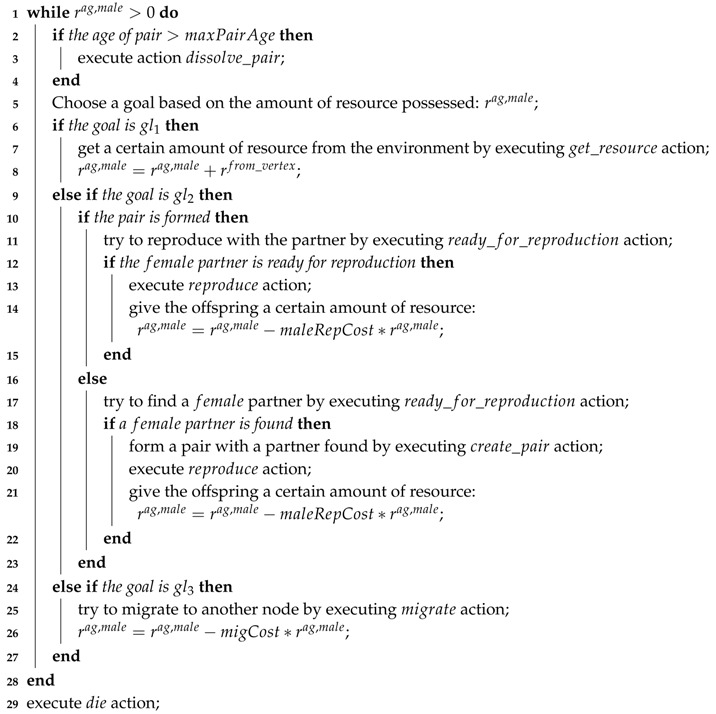

### Appendix A.4. Relations

The set of relations between agents is defined as follows:

(A12) $$Rel=\left\{\overset{\left\{get\_resource\right\}}{\underset{\left\{get\_resource\right\}}{\to }},\overset{\left\{choose,create\_pair,reproduce\right\}}{\underset{\left\{ready\_for\_reproduction,create\_pair,reproduce\right\}}{\to }},\overset{\left\{reproduce\right\}}{\underset{\left\{ready\_for\_reproduction,reproduce\right\}}{\to }}\right\}.$$

The relation representing the phenomenon of competition for limited resources between agents is defined as follows:

(A13) $$\overset{\left\{get\_resource\right\}}{\underset{\left\{get\_resource\right\}}{\to }}=\left\{\langle Ag^{\left\{get\_resource\right\}},Ag^{\left\{get\_resource\right\}}\rangle \right\}.$$

$Ag^{\left\{get\_resource\right\}}$ is the set of agents that can execute get_resource action.

The relation that represents the phenomenon of sexual selection is defined as follows:

(A14) $$\begin{array}{cc} \overset{\left\{choose,create\_pair,reproduce\right\}}{\underset{\left\{ready\_for\_reproduction,create\_pair,reproduce\right\}}{\to }}= & \{\langle Ag^{female,\{choose,create\_pair,reproduce\}},\\ & \;Ag^{male,\{ready\_for\_reproduction,create\_pair,reproduce\}}\rangle \}. \end{array}$$

$Ag^{female,\{choose,create\_pair,reproduce\}}$ is the set of female agents that can execute choose, create_pair and reproduce actions. $Ag^{male,\{ready\_for\_reproduction,create\_pair,reproduce\}}$ is the set of male agents that can execute ready_for_reproduction, create_pair and reproduce actions. A female agent chooses a partner for reproduction from male agents that are ready for reproduction. Next, a pair is formed (create_pair action is executed by both agents) and the reproduction takes place (reproduce action is performed by both agents).

choose action is used by a female agent to choose a male agent from the set of agents that are ready for reproduction (ones that executed ready_for_reproduction action) and are located within the same vertex as the given female agent. When there is more than one male candidate, then the choice of female agent is based on the similarity of female’s preferences and male’s features. The more similar a female agent’s preferences (encoded within its genotype) and a male agent’s features (also encoded within its genotype) are, the more likely a given male agent will be selected.

The following relation represents interactions between male and female agents when a pair is formed, and both agents are ready for reproduction:

(A15) $$\overset{\left\{reproduce\right\}}{\underset{\left\{ready\_for\_reproduction,reproduce\right\}}{\to }}=\left\{\langle Ag^{female,\{reproduce\}},Ag^{male,\{ready\_for\_reproduction,reproduce\}}\rangle \right\}.$$

$Ag^{female,\{reproduce\}}$ is the set of female agents capable of executing reproduce action. $Ag^{male,\{ready\_for\_reproduction,reproduce\}}$ is the set of male agents capable of executing ready_for_reproduction and reproduce actions. A male agent announces that it is ready_for_reproduction and, if a female agent that belongs to the same pair is also ready for reproduction, the reproduction process takes place—both agents execute reproduce action.
